# Medicine, Pathological, Practical, and Therapeutical

**Published:** 1836-01

**Authors:** 


### MEDICINE,

### PATHOLOGICAL, PRACTICAL, AND THERAPEUTICAL.

### *On the Plague now prevalent in Egypt.* By Clot-Bey.

[The following particulars of the plague are communicated by Clot-Bey, in a letter to Dr. Chevrin, dated Cairo, the 26th March, 1835, from his actual observation. There are but twenty physicians at Alexandria and Cairo; the greater part are ultra-contagionists, who cover themselves with waxed cloth, use long sticks, and examine their patients at some distance: others, who are less timid, merely avoid touching the sick,—feeling the pulse through a tobacco-leaf, or after having soaked their hands in oil or vinegar. Clot-Bey himself, and one Spanish and three French physicians at Cairo, and two Frenchmen at Alexandria, use no precautions, and examine the dead bodies very carefully.]

The plague commenced in Alexandria in November; for a month it was very fatal, and altogether 20,000 persons have died. It began in Cairo in December, but during the last fortnight only it has been of a serious type. The first symptoms are pain in the head, nausea, and vomiting, injected eyes, staggering walk, as if from drunkenness, stupid expression, white moist tongue, full and frequent pulse. At this period emetics and diffusible stimuli may be tried, but Clot-Bey knows nothing of their effect. On the second or third day there is mental confusion, sometimes delirium; the tongue is dry in the centre, with red edges; the skin hot; there is often pain in the epigastrium; rarely diarrhœa. Buboes and carbuncles. There is now actually irritation in the digestive canal, brain, and lymphatic glands, and bleeding and cupping are employed, with cauterization to the buboes and carbuncles, to fix this irritation in the skin. On the fifth and sixth day, petechiæ and blue patches on the skin. Revulsives to the extremities. This treatment has apparently saved some patients. The corpses have not the hideous aspect which physicians have described and artists painted. The petechiæ are particularly on the neck, sides of the chest, and limbs; the buboes in the groins and arm-pits, very rarely in the neck; all the lymphatic glands were enlarged in those who had no buboes; carbuncles in three cases only. No particular tendency to rapid decomposition; subcutaneous veins not apparent; heart and veins in the cavities gorged with black blood, as well as the liver and spleen; this viscus was generally doubled in size and softened; arteries empty; kidneys of a deep violet, gorged with blood, hemorrhage in their pelves; the stomach always contained a blackish fluid; its mucous membrane, much injected, exhibited red patches like petechiæ, which sometimes, from their size, might be called ecchymoses; their last degree is ulceration: the intestines were in a similar condition, but less well-marked; the lymphatic glands were always engorged, sometimes increased five or six times, softened, and of a colour like lees of wine, and sometimes black; those of the groin, or arm-pit, by their agglomeration, formed a homogeneous mass of a colour almost always like lees of wine, with effusion of black blood into the surrounding cellular tissue: a similar change was seen in the chain of glands along the vessels of the abdomen and chest; and in many cases the extravasation of blood around them amounted to hæmorrhage. Sub-arachnoid veins and the sinuses gorged: parenchyma of the brain and spinal marrow natural, except in two or three cases, where it was softened.

[Clot-Bey, who is evidently an anti-contagionist, admits that a letter is not the vehicle for the discussion of so important a question. He has observed some facts tending to prove transmission; but how many (he says) are there against it? The physicians, students, and nurses, who were in constant contact with the sick, had not at the date of his letter suffered. The disease began in November, in Alexandria; the first cases in Cairo were in January; and the communication was free between Rosetta, Damietta, which were not affected, and Cairo, where it only arrived after two months. Isolation in houses and ships has not prevented it. The poor are the greatest sufferers, and particularly the Maltese, who are the dirtiest, and whose temperament approaches that of the Arabs. Neither inundations, nor the bad system of burials, explain it; as great inundations have occurred during the last ten years, and there was no deficiency of burials; nor were they more hastily performed than in 1831, during the prevalence of cholera.]

*Journal Hebdomadaire des Progrès des Sciences Médicales,* Juin, 1835.

---

### *On the Effects of Nux Vomica and Aconite on Animals.* By Dr. LOMBARD, of Geneva.

DR. LOMBARD, while employing these remedies in his clinical practice, thought of trying their effects on animals, and made some experiments accordingly, chiefly in reference to their action on the heart. The experiments were performed on frogs, whose hearts beat with great regularity, and for a considerable time after the animal has been mutilated. The medicine was introduced into the stomach, or applied locally to the heart, which was laid bare after the animal had been stupified by blows on the head.

1. *Nux Vomica.* When introduced into the frog's stomach, it produced tetanic convulsions, which in a few hours caused death. The contractions of the heart were sometimes strong and complete, sometimes irregular, tumultuous, and intermitting; always diminished in frequency. Applied locally to the heart, it slightly stimulates, rendering the pulsations more energetic and frequent.

2. *Aconitum.* Its internal employment renders the pulsations less frequent, without irregularity. It has, therefore, a decidedly sedative effect on the heart.

From these experiments, M. Lombard ventures to predicate the effects of the same remedies on the human body, and thence deduces the following practical inferences:

1. That nux vomica cannot be used with advantage in any diseases of the heart; for, although it diminishes the frequency of the pulsations, it renders them irregular.

2. That the aconite, being decidedly sedative, is a proper remedy in active diseases of the heart.

This effect of aconite has been observed on the human subject. In cases of poisoning, the contractions of the heart have been found diminished, and almost suspended. (Orfila, *Toxicologie,* t. ii. p. 221.) It is also considered by the homœpathists to be an energetic antiphlogistic. On these grounds, M. Lombard has tried it in acute enteritis, pneumonia, &c. with success, and also in hypertrophy of the heart. He has often found some drops of the tincture diminish the frequency of the pulse, but more frequently diminish its strength and hardness.

*Gazette Méd. de Paris,* Oct. 10, 1835.

---

### *Clinical Report of the Academical Years* 1832-33 *and* 33-34. By G. DEL CHIAPPA, Professor of Clinical Medicine, Pavia.

[THE only part of this long report which we shall analyze, relates to the general character of the diseases, or to what is called the epidemic constitution, a very important object of medical research. In selecting Dr. Chiappa's paper for publication, we are not solely influenced by its value, but partly also by the wish to illustrate the state of medicine as widely as we can in foreign countries. While it will be found that we have availed ourselves, to a great extent, of the French and German journals, it will probably be remarked that our extracts are much more sparing from the

Italian. This is, no doubt, partly owing to the greater difficulties we have met with in obtaining the latter; but there is another cause, the greater rareness of papers of value in the periodical literature of Italy. For instance, the present is the only thing we could cull from a volume of upwards of four hundred pages, constituting the Numbers of Omodei's Annals for July and August of the present year.]

About the year 1802 (says Prof. Chiappa) a constitution of sthenic diseases commenced in Italy and in all Europe, and has continued to the present time. It has increased, decreased, and become variously modified, sometimes preferring one form, and at other times another (such as gastric, bilious, rheumatic, &c.); but notwithstanding these variations, the general character of disease has always been phlogistic. The treatment required has been the antiphlogistic, modified according to the various circumstances. The principal changes since 1802 were in 1805-6, when the constitution was rather asthenic; in 1809, 10, and 14, when it was strongly phlogistic; in 1816-17 when it inclined to the nervous, continuing more or less phlogistic, with a prevailing disposition to rheumatic and catarrhal, or gastric and bilious diseases; and in 1832-33, when all the diseases manifested a nervous character. In 1834 the constitution became eminently phlogistic, the diseases requiring very full and frequent bleedings. The prevailing diseases were inflammation of the viscera of the head, chest, and abdomen; and numerous fatal cases of puerperal fever. In this phlogistic constitution the sanguineous system was principally affected. The signs of this were, strong pulsations of the heart and arteries, most intense fever, blood covered with thick, coriaceous buff, the inner surface of the arteries a deep red, and as if varnished; and large fibrinous clots in the auricles and ventricles, large vessels and small branches: symptoms of encephalitis, pneumonia, and abdominal inflammation in the same subject, and traces after death of inflammation in more than one organ, and often in all.

During the continuance of this constitution, the mortality was considerably increased. Thus in 1833-34 it was seven per cent.: 316 patients having been admitted and 14 died; whereas in 1832-33 but two per cent. died; there being but three deaths and 150 cases. There was also an increase in the mortality of the whole population of Pavia and its suburbs, which would have been still higher had there not been fewer deaths from chronic diseases, as the winter was unusually mild. It was observed that those who had previously any organic disease, particularly of the circulatory system, when attacked with inflammation, either died or were in the greatest danger.

[The pathological value of the intense red colour of the inner coats of the arteries is a subject of uncertainty, and the cause of much difference of opinion. Dr. Chiappa attributes the redness in his cases to the state of the blood during the general inflammatory condition of the whole system, depending on the epidemic constitution of the seasons. In one case, the particulars of which are given, he states, that besides large masses of coagulable lymph in the cavities of the heart, and cylindrical or conical concretions in the arteries, the coloured part of the blood was deposited, by means of the coagulable lymph, on the inner surface of the arteries, from which in many places it could be removed in small layers by the point of a scalpel. The redness thus depending rather on a varnish spread over the inner surface, than on an injection of its vessels. Rasori first observed this, and Dr. Chiappa confirms it. (This colour of the arteries may probably be produced by many causes. The intensely deep red stain which is found in some fevers, where the blood is almost in a state of putridity, and quite fluid, must be owing to a condition the opposite to that described by Dr. Chiappa.) Several very long cases are given; we shall condense one, as well from its illustrating Dr. Chiappa's views, as from its intrinsic interest.]

S. L. æt. 38, an agricultural labourer, robust, sanguineous, and addicted to wine, suffered much from domestic miseries; for two or three years his health had declined, and he was affected with a number of complaints generally classed under hypochondriasis, such as headach, vertigo, wandering pains in the chest, loins, and extremities; flatulence, dyspepsia, anorexia, or at other times excessive hunger; bowels sometimes constipated, sometimes relaxed; piles, ardor urinæ, palpitations of the heart; occasional febrile attacks, &c. He had consulted during this time all the medical men of his neighbourhood, who must have considered his disease as essentially inflammatory, for they had bled him 30 times. But he never gained by treatment more than partial and temporary benefit. When he entered the hospital he had inflammation of the right testicle, which was enormously enlarged, and accompanied with a deep fistulous ulcer of the scrotum. There was a fistulous ulcer above the left eye, which discharged pus constantly. Pulse hard, and tense; occasional but infrequent febrile disturbance. Like other hypochondriacs he daily complained of new diseases and pains in other situations, and particularly of uneasy and uncomfortable sensations, which are endured with difficulty, and indicate a proportionate lesion of the sensorial power. Antiphlogistic treatment was employed, and chiefly bloodletting, and the blood drawn was sizy. Other antiphlogistic remedies were prescribed, such as cathartic neutral salts, nitre, tartar emetic, digitalis, &c. The aqua lauri cerasi was tried, but without advantage. Bloodletting was performed in all eighteen times, and the blood continued to present the same appearances. The disease indeed appeared to yield, the symptoms were more or less quieted or removed, the testicle diminished gradually towards its natural size, and the ulcer healed; but the various organs were so deeply involved, that a cure was beyond the powers of art. He died, forty days after admission, in a lethargic state, which had been preceded by long and obstinate wakefulness and slight delirium.

The examination of the body was performed with great care by that experienced anatomist, L. Ciniselli of Pavia. Beneath the integuments of the head was a purulent sinus, opening above the upper eyelid and extending to the temporal region, with necrosis of the corresponding extremity of the great ala of the sphenoid bone. Many vessels distended with blood passed from the cranium to the dura mater, which was enormously indurated, and adherent in many places to the arachnoid. This membrane was opaque, thickened, covered with white spots of lymph, and contained much limpid serum. The hemispheres of the brain were adherent inferiorly, and the pia mater every where minutely injected. The substance of the brain was excessively hard, its vessels more numerous and larger than usual; the lateral ventricles full of limpid serum, and the plexus choroides unnaturally developed. The tuber annulare was intensely red, externally and internally, and the inflammation extended to the substance of the peduncles of the cerebrum, as far as their entrance into the hemispheres, to the medulla oblongata, peduncles of the cerebellum, especially the left, entering with it into the hemisphere, around which point the cerebral substance was reduced to a pulpy state for half an inch square. The whole cerebellum was more or less inflamed, externally and internally, and chiefly on the left side. Pus was found beneath the dura mater of the spine, about the last cervical vertebra, and at the termination of the medulla, originating from caries of the fifth and sixth cervical, last three dorsal, and first two lumbar vertebræ, by which their bodies, and the intervertebral substance of the cervical, were destroyed; deep purulent sinuses passed from these into the deep muscles of the neck, and to the viscera of the thorax and abdomen: the dura mater corresponding to the caries was a deep red, the medulla beneath an unformed pulpy mass: above this the opaque arachnoid was distended with serum, and the spinal marrow extremely inflamed over all its surface: the last cervical and dorsal nerves, for an inch or two, presented the marks of intense inflammation. The lungs were universally and strongly adherent to the costal pleura, and the pulmonary pleura was one or two lines in thickness. The right lung was full of small abscesses, as well as the upper lobe of the left; and its inferior lobe congested with blood. The pericardium was adherent externally to the surrounding parts; between it and the heart was a considerable mass of yellow cheesy matter, in greater abundance at the base, and penetrating between the fibres of the heart itself. The heart was not altered, but contained polypiform concretions. The diaphragm was totally adherent to the lungs and heart. The liver was very large, yellow, and, as well as the gall-bladder and duodenum, full of bile. The spleen enlarged, and adhering by plastic lymph to the parts around. The portal system gorged with blood. The pancreas large, and unusually hard. The stomach placed vertically and contracted; its parietes very thick, and the pylorus indurated; many lumbrici in the intestines.

The right kidney enlarged, of a dirty grey colour, very easily broken up. The right testicle three times the size of the other, its tunics inflamed and thickened, and an abscess with an hydatid in the epididymis. The right spermatic cord enlarged, vas deferens dilated and containing pus. The right vesicula seminalis converted into an abscess, which extended into the prostate. The cartilage of the right sacro-iliac symphysis destroyed, and caries of the articular surfaces of the ilium and sacrum, from whence pus escaped into the left iliac fossa.

[In this singular case (which is without exaggeration called a compendious pathological museum), there was neither headach, cough, marked dyspnœa, nor pyrexia, except slight and seldom. Dr. Chiappa imagines that the disease commenced by inflammation of the sanguiferous system, in which all parts necessarily participate, and from thence was diffused through all the internal parts of the body. During the high inflammatory constitution of the year 1834, the chronic affection became acute, which led the patient to enter the hospital, where he died.]

The constitution of 1832-33 was of a nervous character. There was a predominance of affection of the nerves in all the forms of disease, and very little abstraction of blood was comparatively necessary. This lesion of the nervous system was met with in all the forms of thoracic, abdominal, and articular inflammations, rheumatic affections, &c. There was also a great prevalence of the neuroses; neuralgia, gastralgia, hepatalgia, hysteria, hypochondriasis, &c. The chief characters were, 1. Very marked slowness of the pulse. 2. Blood not sizy, but liquid and very soft, the crassamentum like the thinnest jelly, rendering the serum turbid and coloured on the slightest motion. 3. Urine thin, crude, approaching a green colour. The diseases themselves were of a particular character, marked by the following symptoms: course of the disease irregular; changeable pains in various parts of the body ; pulse full, but unresisting; one pulse varying from another; frequently palpitations of the heart; tongue generally foul, bowels usually sluggish, right hypochondriac, umbilical, and epigastric regions frequently so sensible as not to bear the slightest touch, or even the weight of the clothes. The same increased sensibility over the chest; painful sensations in the false ribs, scrobiculus cordis, upper part of sternum, &c.; deep and frequent sighing: sometimes the patients feel well, at other times altogether indisposed; sometimes they sleep, and at others pass the night in constant wakefulness; they are now in good spirits, and shortly after depressed and silent; at one time the appetite is good, and then altogether absent. It is a singular circumstance that these patients do not visibly decline in appearance, and even after a long illness they are found abundantly fat. The mortality in the hospital during this year was only two per cent.

[It seems now probable that a mitigated humoral pathology will be founded on the basis of pathological anatomy, the cultivators of which were for a considerable time determined solidists. M. Chiappa's observations on the state of the blood in various epidemic constitutions are worthy of attention, whatever importance may be attached to the particular medium through which he views his facts. The condition which he calls inflammation of the whole sanguineous system would be more commonly called Plethora, as it is difficult to conceive inflammatory action of such extent, and involving so important a system, without more intense symptoms. Dr. Barlow, however, who has paid such close attention to the general condition of the body in the earliest deviations from health, uses the term constitutional inflammation to indicate a general inflammatory action in the system unattended by local inflammation: a condition marked by hard and quick pulse, white tongue, and increased heat of skin, which may continue for months or years without any specific disease ensuing, and with only progressive deterioration of the general health. The blood too is cupped and buffed, and antiphlogistic treatment alone affords relief.* The existence of this condition, as the effect of an epidemic constitution, is not remarked by Dr. Barlow, and he differs from our author in attributing the inflammatory action to the whole system, not to the circulatory system. Whether this condition

* Cyclopædia of Practical Medicine. Art. *Plethora.*

can with strictness be termed inflammation is a question; there can however be no difference of opinion as to the treatment.]

*Annali universali di Medicina. Luglio ed Agosto,* 1835, vol. 75.

---

*On the Pneumonia of newly born Children.* By Dr. Kluge, of Berlin.

During the cold autumn of 1817, Dr. Kluge first observed this complaint in two children, who both died at the hospital of "Charity" at Berlin. He had many other cases during the following severe winter, but from recognising the first symptoms, he lost but three cases out of a large number. The characteristic symptoms which appear before those of pneumonia are coldness, paleness, and an ash-grey or lead colour of the skin, as in the commencement of cyanosis. The dyspnœa is not observed until this colour has become well marked. Dr. Kluge considers this disease to be owing to some impediment in the pulmonary circulation, producing a reflux of venous blood into the arterial, through the foramen ovale, which causes congestion of the lungs, and death by suffocation. If the change of colour in the skin is observed before the dyspnœa sets in (and it precedes it by many hours, sometimes twenty-four), the patient may be saved by two leeches to the sternum, and calomel; but when the dyspnœa has commenced, there is generally no hope. It is most prevalent in February, especially if the cold increases suddenly and considerably; it is less frequent in April and October. Some years may elapse without a case; but as it appears suddenly, the first attacked are often lost; under such circumstances Dr. Kluge attentively watches for the first symptoms, and the greater part of the other cases are saved. The youngest are most liable to be seized, and it has not been observed in any children above six weeks old. This is explained by the foramen ovale and ductus arteriosus beginning to close about this time, for they remain open during the first six weeks. Rudolphi had asserted the contrary, but Dr. Kluge convinced him he was in error by numerous examinations of newly born infants who died at his hospital. The disease is clearly illustrated by two cases.

*Case* 1. L. B. æt. 29, was delivered after a natural, but rather protracted labour, of a vigorous, well-formed male infant. Twenty-four hours after birth the following symptoms were observed in the child. Extreme paleness of the skin, agitated motions, sharp interrupted screams, disinclination to remain at the breast. In half an hour the skin changed to a grey lead colour, with a mixture of blue, particularly round the mouth and nose, where the colour was deeper; and in an hour from the commencement of the attack, the increase in this colour denoted some obstacle to the performance of respiration. The infant's cries became more choked; it uttered interrupted moans, with a constant, short, dragging cough, sometimes coming on in paroxysms, and requiring strong exertion. The pulse, when compared with that of other infants, was very rapid, and felt with difficulty. Temperature of the skin diminished. One leech was applied to the upper part of the sternum, and bleeding kept up for half an hour; one grain of calomel, with two grains of magnesia, was given internally. These remedies were apparently beneficial, the respiration becoming more easy and free, and the colour of the skin less deep, but an hour and a half afterward the breathing was again more laborious and frequent, the colour of the skin deeper, (on the face almost black,) the breath and extremities cold. Death from asphyxia six hours after the beginning of the attack.

*Case* 2. A male infant was born at half-past twelve at noon in a state of asphyxia, from which he recovered in a quarter of an hour, after allowing an ounce of blood to escape from the funis, sprinkling him with cold water and baths. At 5 p.m. his face and hands were observed to be of a leaden colour; shortly after, diminished heat of skin, dyspnœa, broken moans, but without cough. One leech was applied and a grain of calomel given, but without advantage, except a temporary improvement of half an hour. Respiration became more rapid, unequal, sometimes intermitting altogether, breath and skin more cold. The lead colour was but little marked, and only increased in depth an hour before death, which took place from asphyxia at 2 a.m. Professor Froriep carefully examined both bodies. Upper half of the body of a violet colour, as well as the conjunctiva, tongue, and gums. Sanguineous congestion of the cellular tissue of the aponeurosis of the cranium; of the sinuses of the brain, and of the brain itself; extravasated blood in the base of the cranium. Veins of the neck and subclavian veins distended. Pericardium containing some red serum. Heart natural, its left cavity contained only a small quantity of blood: on the contrary, the right cavities were considerably gorged, as well as the trunk of the pulmonary artery. Foramen ovale and ductus arteriosus open. The greater part of the lungs (except some patches of a bright red) of a violet colour, almost approaching brown, and these parts sunk in water: in some places exudation of a brownish serosity between the pleura and lungs. All the veins in the posterior part of the thorax gorged with black blood.

[Dr. Kluge's explanation of the cause of the discoloration of the skin, &c. in these cases, is ingenious, and agrees with the opinion first satisfactorily proved by M. Louis, and since generally admitted, that the morbus cæruleus is not owing merely to the foramen ovale being open, but that there must exist at the same time an obstruction to the passage of blood out of the right side of the heart. The obstruction is in this case in the lungs, and as the foramen ovale is open, a mixture of the red and black blood takes place. The cause of the first impediment to the circulation through the lungs is not explained; when, however, a mixture of the two kinds of blood has once taken place, pulmonary congestion would follow, and still increase the impediment. The leaden discoloration of the skin of infants (independent of that produced by laborious deliveries), has been mentioned by Dr. Underwood.* He distinguishes it into two kinds; one which entirely goes off and returns, and is commonly dangerous, and which he states (somewhat loosely) depends on some internal mal-formation or derangement, as he has found on examination; and a more innocuous kind, probably depending "on some spasm affecting the external veins, and interrupting the free return of the blood into the larger vessels." No other symptoms are mentioned, and it is therefore very possible that the diseases described by Dr. Kluge and Dr. Underwood are dissimilar. As far as diagnosis is concerned however, it is important to know that a milder disease with the same prominent symptom may exist; and as Dr. Kluge lays it down as a rule, that unless remedies are applied before dyspnœa is observed, and when the colour can alone guide us, there is but little or no hope, it is satisfactory that both writers agree as to treatment. Thus Dr. Underwood advises one or two leeches as the most successful remedy. He previously recommends stools to be procured by clysters; vomiting to be excited if the infant appears sick, and gentle friction before the fire; all of which must be useful adjuvants to leeches in the serious disease described by Dr. Kluge.]

*Medizinische Zeitung*, Berlin, Juli 29, 1835.

---

*Pathology of Rheumatism.* By M. R. Parise.

[This paper is written to prove that rheumatism is an irritation either of the large nervous trunks, or their ramifications, or their intercellular expansions, or the fibrillæ running among the muscles; that is, one of the neuroses. The author does not include articular rheumatism, which he regards as a simple inflammation of the membrane lining the joints. He has suffered from rheumatism in his own person, and as this does not deprive the sufferer of his mental powers, or interfere with their activity, we may admit this claim to attention. His arguments are—]

1. That pressure does not increase the pain, but often diminishes it. It is striking how the slightest motions of a limb may increase the pain, whilst compression relieves it.

2. It does not terminate in appreciable organic lesions, even though it should last for months or years. The muscular and nervous tissues are unchanged: there is never suppuration, and it is doubtful whether serous or gelatinous effusions beneath aponeuroses, or the sheaths of tendons, is the result of rheumatism which has preceded them.

3. Its mobility is a distinguishing character.

4. Its change of situation does not change its nature, although it goes by different

* On Diseases of Children, 9th Edition, p. 115, et seq.

names. Thus it is the same disease which in the head is called *gravedo*, in the neck *torticollis*, (wry-neck,) in the side *pleurodynia*, in the loins *lumbago*, and along the sciatic nerve *sciatica*.

5. Rheumatic pains are more severe in the night, which is a character of neuroses. Heat cannot explain the fact, for the same heat during the day has no such effect. The cause is hidden.

6. Rheumatic parts are affected constantly by changes in the air and by electricity; the sufferers are living barometers: and this can be explained by the anormal sensibility of the nerves.

7. The distinguishing characters of the neuroses are, the irregularity of their progress, a tendency to periodicity, a disposition to cease and to return rapidly. These features distinguish rheumatism. We shall offer no apology for not translating the following pertinent jeu d'esprit from one of Madame Sévigné's letters to her daughter, as it is too "spirituel" to bear translation. "Devinez ce que c'est que la chose du monde qui s'en va le plus vite et qui s'en va le plus lentement; qui vous fait approcher le plus près de la convalescence, et qui vous en retire le plus loin; qui vous fait toucher l'état du monde le plus agréable, et qui vous empêche le plus d'en jouir; qui vous donne les plus belles espérances, et qui en éloigne le plus l'effet; ne sauriez-vous le deviner? Eh bien! c'est un rheumatisme."

8. The facility of return after its apparently complete cure is so constant, that adults, and still more old people, never are sure, after having been several times affected, that they are permanently cured. Hence the rheumatic diathesis. Young people are more often completely cured; which is probably owing to their perspiring more freely and generating more heat, from a more active capillary circulation. This becomes more languid in advancing years; and explains the greater tendency to rheumatism in the old; in different seasons and climates; and the action of flannel, rubefacients, &c. in its cure.

9. Finally, it is often cured by antiperiodics, as bark; but M. Parisé proposes to discuss its treatment on other occasions.

The obscurity which hangs over rheumatism renders every attempt at its elucidation recommendable. The similarity between rheumatism and neuralgia is clearly stated by M. Parisé, but his opinion that articular rheumatism is a pure and simple inflammation of the membrane lining joints, going through the usual stages of inflammation, and requiring a similar treatment, is too hasty an assertion. If this were the case, there would be no difficulty in the matter; but the circumstances of its not yielding, like other inflammations, to treatment, its not producing the same rapid disorganization, its rapidly shifting its situation, the facility of its return, and the difficulty of a complete cure, mark its specific nature, and have led pathologists to class it with the affection which M. Parisé has described.

*Bulletin général de Thérapeutique*, Juillet, 1835.

---

*On the Employment of Nux Vomica and its Preparations in the Treatment of Dysentery.* By E. GEDDINGS, M.D.

[ALTHOUGH there is nothing particularly novel in the following observations, yet as forming a slight contribution to the very important department of therapeutics, we think them well deserving of attention. They are from the pen of an experienced American physician.]

Foiled of success, (says Dr. Geddings,) and discouraged by the general result of all these [the more ordinary] remedies, we have been induced, within a short time, to make trial of the nux vomica, in some cases of this disease, [Dysentery.] We have as yet only had an opportunity of using it in a few instances, and those not of the worst character, yet so difficult to manage, that some of them had previously resisted all our remedies. The cases selected were those which were not attended with much febrile excitement, but which were characterized by frequent calls to stool, considerable griping and bearing down, and an inability to pass any thing but mucus, or that streaked with blood. In some of the cases, the remedy, though certainly beneficial, was not competent alone to accomplish a cure; in others its good effects were so striking as to inspire considerable confidence in its virtues, and to induce us to make this notice, with the view of inciting others to give it a fair trial under similar circumstances. We do not wish to recommend it to the exclusion of other means, or to inspire a hope that it will be found capable of itself of curing the disease in a large number of cases. But from what we have seen of its effects, we feel assured that it will be found a useful adjuvant, and that in some cases at least it will afford relief when other remedies fail.

We commenced at first by administering the nux vomica in powder, in doses of seven grains, three times a day, as recommended by Vaux of Ipswich, England.* In one individual to whom the article was administered in this form, the good effects were prompt. The griping, tenesmus, and frequent calls to stool were speedily checked; the discharges became natural, and the patient, who had suffered much, and had failed to obtain relief from the treatment previously prescribed, expressed himself delighted with the remedy. It was also beneficial in other cases, as were the alcoholic extract of Pelletier, administered in doses of two grains, three times a day, and the strychnia, given in form of an acetate, in doses of one twelfth to one sixth of a grain, formed by dissolving the strychnia in acetic acid. Our comparative trials of the different preparations have as yet been too limited, to enable us to decide which deserves the preference; but we are inclined to prefer the powder, and next to that the extract. It will perhaps be beneficial to combine with whatever form is employed, a small quantity of opium, or some of its preparations.

The good effects of nux vomica in several of the affections of the mucous membrane of the digestive organs, have long been known. Hagström, a Swedish physician, was, we believe, the first who recommended it in dysentery, and his testimony in its favour was of the most flattering character. The celebrated Hufeland † states, that he derived great benefit from it in the treatment of epidemic dysentery; and Thomann ‡ remarks, that he has seen it effectual in allaying the tormina, and abating the inclination to go to stool. Richter § observes, in reference to the efficacy of this remedy in dysentery, that the extract, like opium, tends directly to allay the irritation of the alimentary canal, and subjoins, that combined with the article just mentioned, it proves beneficial where opium alone fails to do so. The following is the form in which he administers it:

℞ Extract. Nucis Vomic. ℈ ss. Mucillag. Gum. Mimos. ℥ j. Aqua Font. ℥ vj. Syrup. Althæ. ℥ j.

M. S. Two table-spoonsful every two hours.

By Most, § a recent writer, this article is especially recommended in what he denominates *pituitous dysentery*, and he remarks, that when the disease is protracted, the article may be administered in the following form for several days in succession, with great advantage:

℞ Nuc. Vomic. ʒ j. Infunde in aqua ferv. q.s. Digere per ½ hor. ut reman. ℥ vj. Col. adde Tinct. Opii Simp. ʒss.

M.S. A table-spoonful every two hours.

By Frisch, a German physician of celebrity, the remedy is highly recommended. He remarks, that in those forms of diarrhœa dependent upon a subacute inflammation of the mucous membrane of the intestines, which are attended with frequent discharges of tenacious mucus, and much griping and tenesmus, no remedy is so effectual as nux vomica.¶ Its efficacy in diarrhœa has also been testified by others. In a case of chronic diarrhœa, in an individual of a nervous temperament, Professor Récamier

* Lectures on the Morbid Anatomy, Nature, and Treatment of Acute and Chronic Diseases, &c. By the late John Armstrong, M.D. &c. Edited by Joseph Rix, M.D. p. 409. Lond. 1834.

† Journal der Praktisch. Heilkunde. Bde. i. stucke 1.

‡ Summa. Observat. Med. tom. iii.

§ Die Specielle Therapie. p. 133, Bde. ii. Berlin, 1821.

‖ Encyklopädie der gesammten medicinischen und chirurgischen Praxis. Thiel ii. p. 317. Leipzig, 1833.

¶ Thompson's Materia Medica and Therapeutics, vol. i.

administered the alcoholic extract of nux vomica, in doses of one eighth of a grain, with complete success, after various remedies had been resorted to ineffectually.*

From these remarks it will be seen, that the remedy is at least deserving further trials. To expect it to perform the part of a specific would be an absurdity, nor would it be reasonable to expect much from it in the acute stage of dysentery. But after suitable depletion, and especially when the disease is verging upon a chronic form, we doubt not it will be found useful.

*American Archives of Med. Science, No.* 2, *Nov.* 1834.

---

*On the Use of Purified Alumina in Infantile Cholera, and of Sulphate of Copper in softening the Stomach.* By Dr. G. E. F. DÜRR.

Dr. Dürr has been in the habit of using the *Argilla depurata,* in the dangerous forms of diarrhœa and cholera, which are liable to attack children, especially during summer, with considerable success since the year 1826. Its action appears to be very different from that of the carbonate of potass or magnesia. It may be given with perfect safety during the first stage of the disease, when on account of the inflammatory symptoms the muriate and carbonate of iron and opium are contra-indicated, and yet where the rapid course of the disease requires decided and active treatment. It has acted successfully in cases where these astringents and also chlorine water have failed; and Dr. D. prefers it to the acetate of lead, which at this early age is rather a questionable remedy. To use the argill successfully, we must give it in pretty large doses, viz. from half a drachm to one drachm, in any proper vehicle, during the twenty-four hours.

The chief symptoms of this dangerous affection, which runs its course in from two to ten days, are profuse vomiting, without any effort, of a sour smelling fluid varying in consistence; in many cases diarrhœa had lasted a whole week, when the first alarm was excited by the sudden appearance of vomiting. Collapse and rapid emaciation of the body followed, with depression of the anterior fontanelle; hollowness of the eyes, paleness, alteration and shrinking of the features, cold extremities, hot occiput, and more or less fever; *agrypnocoma,* or a lethargic state without actual sleep, restlessness, crying, whining, throwing itself from one arm of the nurse to the other, drawing up the feet to the abdomen, want of appetite, great thirst, stiffness in the nape of the neck, and the stomach so distended that it projected in the left hypochondrium like a distended bladder.

Dr. Dürr's practice in this disease was to quiet the irritation in the stomach and bowels by emollient oleaginous remedies in combination with the argill: to excite the activity of the skin by extr. cicutæ internally, and the application of an epipastic powder externally. The immediate effects of this were diminished frequency of the evacuations, the natural yellow colour returning, quiet, excoriations in the folds of the skin about the neck and groins. In very young children the cerebral affection was often allayed merely by the chlorine water (aqua oxymur.); in older children, or where the symptoms were more violent, by leeches to the scrobiculus cordis, or behind the ears, according to circumstances; the dryness of the skin, the lethargy, and coldness of the extremities, were treated with baths of chamomile and salt, and with cold lotions to the head; warm stimulating aromatic fomentations were used from time to time, and enemata of elder and linseed, to which the yolk of an egg rubbed down with linseed oil was added. Dr. D. assures us that in other acute diseases also, where the rough dry state of the skin had defied the usual remedies, gentle perspiration had followed the use of these enemata. The epispastic powder which he mentions, was first described by Autenrieth; it consists of fresh prepared mezereon bark powdered. When the skin is not very delicate, it not unfrequently fails to produce any effects. Dr. D. has used it combined with calomel, and in very severe cases combined with corrosive sublimate, with great certainty and effect. The spot to which it is applied usually becomes red in the course of from six to twelve hours, and in about as much more time moist and excoriated. If the powder will not stick, he moistens the spot with a little saliva or lard.

The result of his practice is decidedly favorable; of 67 children from the time

* Richard, Elémens d'Histoire Naturelle Médicale, tome ii. p. 148.

of birth to the age of fifteen months which he has treated for this disease during 1833 and 1834, he lost only seven. Dr. Dürr has given several interesting cases, both successful and unsuccessful, together with the examinations of the latter after death. Great congestion of the cerebral vessels, and considerable softening of the stomach, so that portions of it were quite pulpy, were the chief features, and in one case there was perforation. We regret that our limits will not permit us to quote more than one of these.

*Acute Softening of the Stomach.*—A weakly but apparently healthy female infant, eleven days old, brought up on saccharum lactis. The parents lost a child at the same age in August 1833, with symptoms of a similar description, in three days. The child sickened rapidly (Aug. 1834), with diarrhœa of clay-coloured evacuations; constant screaming, restlessness, red tongue, hot skin, distended stomach. ℞ Ol. Amygdal., Mucilag. Acaciæ, āā ʒ ij. Syrupi Altheæ ℥ ss. Argillæ depuratæ, ʒ ss. Aq. Lauro Cerasi, ℈ ss. M. sumat cochl. min. j. quâque horâ. The child recovered.

In the course of a week the same symptoms returned, with aphthæ and erythematous eruption over the whole body; the same emulsion was ordered with the addition of extr. cicutæ, gr. j. The child again recovered entirely. When about three weeks old (Aug. 24,) the diarrhœa returned with vomiting, drawing up the feet to the abdomen, screaming, restlessness, paleness of the face, and the eruption becoming fainter; the same mixture was ordered with the addition of Extr. Cicutæ, Rad. Ipecac. āā gr. j. Enemata, aromatic fomentations. Unguent. Cantharid. behind the ears, and the strong epispastic powder between the folds of the skin in the neck, axillæ, &c.

2*d day.* Little alteration during the night, except that the diarrhœa was not so frequent, eruption still scarcely visible. Stiffness in the spinal column; extension of the limbs; five green spinach-like evacuations; hands and occiput hot; eyes somewhat sunk; anterior fontanelle slightly depressed; slight convulsions; a half moribund look, alternating with screaming, during which the eyes become a little more animated.

℞ Mucilag. Acaciæ, Syrup. Altheæ, āā ℥ ss. Argillæ depuratæ, Liq. Ammon. Acet. āā ʒ ss. Pulv. Ipecac. gr. j. Ext. Cicutæ, gr. ij. M. sumat cochl. minimum quâque horâ, contin. omnia.

3*d day.* Better during the night; bowels opened four times, yellow, not so watery; eruption again visible on the hands; skin moist; excoriations behind the ears; the neck and axillæ are merely red, not yet moist; eyes more lively, less restlessness; symptoms of ulceration in the mouth. Pergat.

4*th day.* Bowels open only once; eyes not so hollow; excoriation of the nates and neck: continue the emulsion, adding to it half a scruple of argilla (in all two scruples.)

5*th day.* The whole appearance, as also that of the eyes, is natural; fontanelle likewise; three yellow evacuations; the soft parts which had been powdered are red: repeat the emulsion only every two hours.

6*th day.* Restless night, again the altered and collapsed appearance; three clayey evacuations: add to the emulsion, Aquæ Cinnamomi, ʒ iij. Extr. Aurant. gr. iv.

7*th day.* Again as on the fifth day, and up to the present time (14th Sept. 1834,) is healthy and thriving.

The two other cases are a complication of the same disease with what the Germans call *Asthma thymicum,* where the child holds its breath when sucking, the nostrils appear stopped up with mucus, and during sleep it suddenly wakes with much dyspnœa and lividity of face. Besides the same treatment as before described, minute doses of cupri sulphas and musk were given. This produced repeated vomiting; the eyes became brighter, the livor of the face disappeared, the cough diminished, respiration was freer, and the child no longer held its breath. We give the formula, as some of our readers may feel inclined to try this plan of treatment in similar cases.

℞ Cupri Sulphatis, Moschi, āā gr. $\frac{1}{8}$. Pulv. Acaciæ, gr. ij. Pulv. Glycyrhizæ, gr.iv. M. Pulveres tales, viij. To take one every half-hour until repeated vomiting has been produced.—*Hufeland and Osann's Journal, B.* 81. *Juli,* 1835.

---

*On Hæmorrhagic Pericarditis.* By Dr. SEIDLITZ, Senior Physician to the Naval Hospital at St. Petersburg.

[THE memoir, of which we here present the reader with an abstract, occupies sixty pages of the last number which has reached us of Dr. Hecker's *Annalen,* and possesses extraordinary interest, as well from the pathological character of its details as from their novelty. We have been as much influenced by a feeling of convenience in adopting the name given above to this affection, as by any strong opinion of its superiority to that employed by our author. In his memoir, Dr. Seidlitz terms the disease *Pericarditis exsudatoria sanguinolenta.* Although we have greatly condensed the whole paper, we believe we have given all that is really important in it of a pathological and therapeutical character; but we have omitted much interesting discussion, in which the author attempts to prove the identity of the disease with the *Morbus cardiacus* of the ancients. For this we must refer the reader to the original article, and to the 31st, 32d, and 37th chapters of Cælius Aurelianus, wherein he treats of the *Morbus cardiacus,* or *Cardiaca passio.* We shall only be able to afford room for a small portion of Dr. Seidlitz's extracts from the ancient author.]

In 1831, several sailors died suddenly at St. Petersburg, whilst engaged at work; others who were admitted at the hospital survived only during a short period. On their bodies being examined by Dr. Seidlitz, it appeared that their death was owing to a severe inflammation of the pericardium, joined to a copious exudation of a sanguineous fluid into the pericardial sac. On his communicating the facts, and exhibiting some of the diseased hearts to the association of German physicians (at St. P.) Dr. Crichton remarked that the disease (which he designated as acute hydropericardia) was one of frequent occurrence amongst the troops. The symptoms were too characteristic to be overlooked on the re-appearance of the disease, and thus Dr. S. was subsequently enabled to treat it with success in its earlier stages, or to verify his diagnosis by cadaveric inspection in the cases which terminated fatally. He characterized the disease as "pericarditis exsudatoria sanguinolenta in homine scorbuto affecto." Its pathological features differed in many respects from those commonly attributed to pericarditis, and it appeared to Dr. S. as a new and peculiar disease. On referring, however, to the description of the *morbus cardiacus* by Cœlius Aurelianus, he became at once convinced of the identity of the last-named disorder with the one he had to treat.

The number of fatal cases of this disease recorded in the hospital books, between March 1831 and September 1834, is stated at fifteen; that of those which terminated favorably was more considerable. Of these cases the majority occurred in February 1832, when the disorder appears to have assumed an epidemic character. It is remarkable that the complaint was only to be met with between the months of February and September; the period during which scorbutic forms of disease commonly prevail at St. Petersburg; and it was usually associated with a transitory epidemic of a rheumatic nature, while pleurisy was commonly prevalent at the same moment. Thus, perhaps, the scorbutic *constitution,* or epidemic tendency to scorbutus, might be considered as the predisposing, the *rheumatic* as the occasional cause of the malady. The disposition to sanguineous exudation from the pericardium was rendered the more evident during those periods, by the fact, that in persons who had died of quite dissimilar complaints, the small quantity of fluid contained in the pericardium had assumed the colour of blood.

*Case.*—Towards the end of February (1831), a sailor, aged 27, of a muscular habit, was seized with an unusual degree of lassitude after a hard day's work. A peculiar sense of anxiety caused him repeatedly to start out of an unrefreshing slumber. Tightness of the chest ensued on the following day. The præcordia seemed the primary seat of distress, from whence an obtuse pain radiated over the whole anterior surface of the thorax, and concentered itself on the second day (that of his admittance at the hospital) in the right hypochondriac region. Both these places were impatient of touch, which without precisely causing pain, seemed to the patient to impede his breathing. The countenance was bloated, of a bilious hue. The breathing was regular, but superficial; deep and full when the patient exerted himself, and attended by neither pain nor cough. Pulse very frequent, and very indistinct (suppressed, unterdrückt). Pulsation of the heart imperceptible to the hand, and scarcely audible. The patient was perfectly conscious, but morose and torpid, preferring to lie flat on his back or on his left side, and with his head low. He denied having been attacked with rigors or with febrile heat. V.S. ad lb. iss. Blood buffed, yielding a large proportion of serum. A solution of nitrate of potash with tartarized antimony, and saline clysmata, were administered. The latter produced several stools. The night was passed more tranquilly, although but with little sleep. The pulse had now become less frequent and more developed, but the pulsation of the heart was more indistinct. The countenance was expressive of great anxiety, but still nothing was complained of but an entire prostration of strength. The temperature of the skin underwent frequent changes from moderate warmth to a marble-like coldness, accompanied by a cold sweat. Action of the kidneys regular. Four cupping-glasses were applied to the region of the liver, and the saline potion and clysmata repeated. On the morning of the fourth day, the patient's apathy had increased: alternately groaning and sighing, he evinced a great dislike to be interrogated. Pulsation, both of the heart and arteries, was no longer distinguishable; the lips were blue; face and extremities of a marble-like coldness. Whilst Dr. S. was in the act of examining the abdomen, the patient suddenly applied his left hand to his head, then to his chest, stretched his eyelids wide asunder, and expired. In the next moment the pupils became strongly dilated. An incision was instantly made into the swollen jugular veins, from which the blood issued in a small stream. Respiration was at the same time artificially sustained by alternately compressing the thorax, and again allowing it to expand, but in vain.

On the thorax being opened, the eye was struck with the extraordinary size of the pericardium, and with its livid appearance, owing to the translucent fluid wherewith it was distended. An incision being made into this membrane, from four to five pounds of fluid escaped, having the colour of dark venous blood and the consistency of a decoction of marsh-mallows. The entire absence of crassamentum disproved this fluid to be genuine blood, whilst the fact of its coagulating through the influence of heat, showed it to consist of the albuminous serum mixed with the colouring matter of the blood. The inner surface of the pericardium was tinged of a dark blue red, and lined with a coat of villous, granular fibres, of the same colour, which lining was easily separated from the pericardium, and had rather the character of a deposit from the fluid than of the false membranes which attach themselves to the pleura in exudatory inflammation of the latter membrane. On removing the above fibrous layer, the envelope of the heart appeared of the same blue red colour; its surface smooth, shining, and sound. The substance of the heart itself was dark red and very firm. The compression by the extravasated fluid had reduced the volume of the heart to the size of a man's fist, and its four cavities to a mere nothing. The abdominal organs were healthy, although the veins were here, as well as in the thoracic cavity, gorged with blood.

The author proceeds to contrast the characteristics of the above case with those of genuine pericarditis, as well the acute as chronic, from both of which its phenomena differ conspicuously, during life and after death. Had the inflammation a scorbutic character? The discoloured state of the heart and pericardium, together with the scorbutic appearance and the rapid collection of the exuded fluid, render it probable. Nor is it disproved by the absence of some of the external attributes of this disease, for it is only in its later stages that scurvy assumes its more characteristic features. This cachexy annually recurs at St. Petersburg, shortly preceding the vernal equinox. The young, plethoric, and robust are first seized with symptoms of inflammatory fever, which last several days, until suddenly—often in the course of a single night—one of the lower extremities assumes a wax-like hardness, together with a blue appearance, from the effusion of serum, mixed with the colouring matter of the blood, into the cellular texture of the skin. Under peculiar circumstances, then, might not such an effusion be substituted by a more copious one from the pericardium ?

In February 1832, notwithstanding the prevalence of w. and s.w. winds, the thermometer remained stationary under 0′, and the epidemic constitution assumed an inflammatory rheumatic character, whilst scurvy was at the same time prevalent. Many instances occurred, not only of pectoral inflammation, but likewise of exudatory pericarditis. A case of the latter is detailed, wherein a more energetic treatment, such as might appear adapted to *acute hydro-pericardia*, led to a successful termination, after about three weeks' illness. The symptoms were here much the same as in the first case, and the diagnosis was confirmed by auscultation with the stethoscope.

About the same period, instances occurred of pericardial exudation *suddenly* supervening upon other affections, and Dr. S. was greatly struck with the following one. A sailor, aged 32, addicted to drinking, and of a lax, bloated habit, entered the hospital on the 20th of February, complaining of pectoral oppression, a humid cough, pains in all his limbs, and in the right hypochondrium. Pulse low and feeble, although not particularly frequent. Temperature of the body natural; tongue furred and discoloured; breath fetid, like that succeeding a debauch. Though appearing to require rest, the patient evinced no real torpor. A solution of the muriate of ammonia with tartarized antimony was administered, and cupping-glasses were applied to the hypochondriac region. After a good night's rest, the patient awoke almost recovered, with the exception of an asthmatic affection, which he declared to be of long standing. The absence of pyrexia, the leucophlegmatic appearance of the patient, the small feeble pulse, and the obtuse sound given by the thorax on percussion, raised the suspicion of a collection of water in the chest, although the patient could lie in any position without inconvenience. He was ordered squills, digitalis, and cream of tartar. The bowels acted spontaneously, and the patient's appetite was very good. Under these circumstances he supped plentifully on the evening of the 22d, and went to bed. Scarcely had he fallen asleep, when he was observed to breathe heavily, and on a sudden to cease breathing altogether. Life had become extinct.

On opening the thorax and abdomen, several pounds of blood flowed away from the two cavities. The right lung partially adhered to the costal pleura, especially towards its inferior lobe, between the extremity of which and the diaphragm a black layer of clotted blood of the size of half a hen's egg was found. The left lung likewise was attached here and there to the costal pleura, but more particularly to the pericardium. The lungs themselves were flaccid, as if macerated. The pericardium contained four pounds of dark blood-coloured fluid without crassamentum. On removing the dark fibrous coat which lined the whole inner surface of the serous membrane, the latter was discovered to be throughout smooth and unimpaired. The heart was enveloped in fat, and compressed into a very small compass; its cavities pressed one against the other, and containing no blood. The jugulars and venæ cavæ were gorged with blood, as were also the vessels of the brain and its sinuses. The liver abounded in blood, and was uncommonly large, but flaccid, retaining the impression of the fingers. The stomach contained the food last taken by the patient, in a perfectly undigested state.

Granting that the pericardium might have contained a small portion of fluid, the deceased's symptoms up to the evening of the 22d appear to Dr. S. wholly disproportionate to the extent of exudation detected after death. He is persuaded that a sudden and copious pericardial discharge succeeded on the patient's falling asleep upon an over-plentiful meal.

At the point of transition from the disease to convalescence, (in the more favorable cases,) the symptoms are usually aggravated to such a degree, that the patients ardently desire to be relieved from their sufferings by death. Nor are the instances frequent where so great a degree of apathy prevails as to render the patient wholly indifferent to his sufferings. An instance is, however, narrated of an almost total paralysis of sensation (so to term it) in a cachetic subject, aged 40. The individual entered the hospital on the 19th of February, with a neglected pleurisy, and was treated accordingly. On the morning of the 25th, the pleuritic symptoms appeared to have vanished, and the patient considered himself better. New symptoms, however, denoted a change for the worse, and pointed to pericardial effusion. The pulse had become low and feeble, and the pulsation of the heart imperceptible to the hand, and nearly so to the ear. The patient's countenance was pale, sunk, and cold; tongue white, and perfectly chill. He had lain during the whole night in a cold sweat, which his bed and body-linen had thoroughly imbibed. Thus he still lay, on his back, unwilling to move. The pectoral pain had left him, and he could draw a deep breath; even anxiety and oppression seemed gone: in short, the only thing at all complained of was heat, with a constant desire for cold drink, notwithstanding the marble-like coldness of the body's surface. He lingered thus until the morning of the 28th.

The results of the cadaveric inspection proved the diagnosis to have been correct in the above case, both as regards the disease in its original and in its metamorphosed shape. Pleural adhesions, superficial inflammation, and hepatisation of the left lung; redness of the parvagum, on the one hand; on the other, distention of the pericardium with three pounds of discoloured serum, together with all the collateral phenomena peculiar to the disease under notice.

The last cases of pericardial disease, during 1832, occurred in May, and were, with two exceptions, successfully treated by the depletory and derivatory methods, joined to the administration of camphor. Convalescence was usually preceded by copious perspiration.

The influenza which raged at the commencement of 1833 created a general disposition to diseases of the thoracic viscera, and late in spring, a scorbutic tendency was superadded. This combination again brought exudatory pericarditis in its train; the first case originating in inflammation of the anterior mediastinum from external injury, and causing death. Two other fatal cases are worthy of being glanced at, the one merely from its complication with nostalgia, (the subject being a native of Poland,) and with severe hepatic disease; the other from its *appearing* suddenly to supervene on intermittent tertian, on which the exhibition of sulphate of quinine, preceded by that of an emetic, failed to have any effect. In both instances the cause of death was again detected within the pericardium.

The tertian fever, connected with the latter of these cases, was *apparently* the original disorder, but it first seized upon the patient under circumstances wholly unfavorable to the generation of intermittent, refusing *nevertheless* to yield to the usual method of treatment. Dr. S. therefore inclines to the opinion that the fever was here merely a secondary affection, a mask assumed by the pericardial disease, such as is not unfrequently observed in organic disorders of the heart. This view seems strengthened by a case of a similar kind which occurred in February 1834, and in which the intermittent paroxysms yielded to a course of diuretic remedies. During this year (1834) the disease attacked the patients less suddenly, and proved less rapidly fatal. The last case that occurred commenced in July, under general symptoms of scurvy, and terminated fatally on the 53d day by a complication of the exudatory pericarditis with jaundice. Dissection exhibited no very novel feature: the pericardium, however, contained no less than six pounds of fluid. The abdominal organs, though tinged with yellow, were otherwise healthy.

It will be seen from the foregoing cases, that the disease rarely admits of a favorable prognosis being formed: where, however, the malady seized upon the patient suddenly, without any premonitory symptoms, Dr. S. never witnessed an instance of recovery.

Amongst the more striking features of resemblance between the symptoms of the exudatory sanguinolent pericarditis of Dr. Seidlitz and the *passio cardiaca* of Cœlius Aurelian, several may be inferred from the following passage (cap. 32) in the latter author: "At vero, si jam fuerit præsens passio cardiaca, sequitur ægros articulorum frigidus torpor, aliquando etiam omnium crurum, vel manuum, aut totius corporis: pulsus densus, celer, parvus, imbecillis, &c.......attestante hallucinatione, animi desponsione, cum vigilibus jugibus, et quibusdam repentino atque concervato per totum corpus sudore. Quibusdam vero primum cervice tenus et vultum, parvus, tenuis, aquætus, dehinc per totum, ut supra diximus, corpus plurimus, ac tunc crassus et tractuosus atque viscosus vel male redolens ......respiratio parva atque anhela et insustentabilis et per morbi progressum rara loquutio ac tremula. Ora pallida, oculi concavi, thoracis gravedo debilitatis causa. Aliquando etiam translatis lingua humecta: aliquibus vero ob complexionem tumoris parvi in visceribus constituti, arida atque sicca, attestante desiderio frigidi potûs. Deficiente ægro, visus obscuritas, articulorum livor, unguium uneatio, et *plurimis integer sensus*," &c.

This author appears to have had some intuitive notion of the *real pathological* nature of the disease. Thus (cap. 34), " alii ex corde, alii ex membrana quæ cor circumtegit diaphoresi vexantur, et propterea ii, qui ex membrana cordis solvuntur adjuncto dolore laborant, qui punctionibus crebris ægrum afficit—ii vero, qui ex cordo, gravedinem solum sustinens."

A long and somewhat intricate analysis of Cœlius Aurelianus's opinions, leaves in the mind of Dr. S. no doubt of the identity of the ancient and modern disease. As, generally speaking (he concludes), the scorbutic diathesis has gradually vanished from Southern Europe, and now occurs exclusively in the Northern zones; in like manner, the *cardiac passion* is at this day only to be met with in these latter regions, where scurvy actually rages with no common virulence.

Cœl. Aurelian's assertion: "phlebotomiam nihil jugulatione differre ratio testatur," does not seem to apply to Russia, where the inflammatory tendency of scurvy usually calls for the detraction of blood. Persons in the habit of being bled annually towards the return of spring, are attacked with the incipient symptoms of scurvy if they delay the venesection beyond the usual period. On such occasions, neither spare diet, refrigerents, purgatives, nor, in fact, any method short of phlebotomy, will suffice to reduce the symptoms. Pursuing the consideration of scurvy through its more advanced stages, Dr. S. pronounces the spots first discovered on the lower extremities to be by no means identical with petechiæ, or sugillations beneath the epidermis, but to be owing to extravasation of blood within the cells, where the capillary roots reside. Hence the skin assumes the appearance and feel of goose-skin. In other cases, the malady localizes itself in the second form,—that of extravasation, in large patches, within the cellular texture of the skin and muscles; or in the third form, producing a wax-like induration of the member. In all these cases, the danger to the central organ of circulation is averted, and the fundamental disease advances in its normal development. Again, the heart is safe when the scorbutic affection, without precisely assuming its ordinary form, attacks other internal organs; as, for instance, the spleen, liver, or lungs. Then arise softening of the spleen, appalling jaundices, (which with their concomitant febrile symptoms present the image of true yellow fever,) or *splenisation* of the lungs. The part of the body in which scurvy locates itself, depends partly upon individuality of predisposition, partly, as has been before stated, on the epidemic character of the period.

*Hecker's Annalen. Zweiter Band. Zweites Heft.* Berlin, 1835.

---

### *On the Use of the Distilled Water of the Prunus Lauro-Cerasus in Facial Neuralgia.* By E. S. BENNETT, M.D.

DR. BENNETT, of Charleston in America, relates some cases, in a late number of the American Archives, successfully treated by the remedy here named. It is to be observed, however, that in two out of the three cases described, belladonna was also employed in combination with the cherry-laurel water. The cases were evidently of the neuralgic character, and of considerable standing and severity; and the relief was speedy and permanent. The mode of applying the remedy was to form a lotion of four ounces of the laurel water, one ounce of sulphuric ether, alone, or with half or one drachm of extract of belladonna; and with this lotion the affected parts, previously covered with carded cotton or cotton-wadding, were kept constantly wet. Dr. Bennett says he could give several more cases of similar character and results, but deems these sufficient to test the virtues of the remedy, which he is assured will be found highly serviceable in relieving the great sufferings of patients similarly affected.

*American Archives of Med. Science, for April* 1835.

---

### *Clinical Observations on the Effects of certain Medicines on the Heart.* By Dr. Lombard, of Geneva.

1. *Assafœtida.* Applied externally in the form of the Emplastrum Galbani comp. (L. P.), assafœtida seldom fails to quiet palpitations. Internally exhibited, it diminishes and renders more regular the movements of the heart, removes palpitations, and produces a state of tranquillity even in those most easily excited.

2. *Camphor.* Given internally, in doses of from three to twelve grains in twenty-four hours, it renders regular the most tumultuous palpitations, and removes the dyspnœa which often attends hypertrophy of the heart with dilatation. An enlarged heart, with obstructed orifices or dilatation, should be regarded as a muscle fatigued by its constant efforts to maintain an equilibrium between the fluids which enter it and those which are expelled from it: hence the indications are, to give it power by iron and quinine, and to regulate its action by camphor and assafœtida.

3. *Digitalis.* Its sedative action on the contractions of the heart is not constant, but seems to depend on the state of the stomach, mode of life, dose, and mode of administration.

(*a.*) When the stomach is irritated, digitalis often increases the rapidity of the circulation. When given to those whose stomachs are very sensitive, but not in an inflammatory state, it often produces vomiting, and the palpitations and frequency of the pulse disappear; if continued, the pulse becomes much slower, and no inconvenience follows but the shocks from frequently vomiting. The effects of the prolonged use of digitalis on the stomach are not to produce inflammation, but rather a particular state of the gastric plexuses of nerves, analogous to that observed in sea-sickness; and it is best obviated by antispasmodics and stimulants; such as subnitrate of bismuth, oxide of zinc, then nitrate or acetate of potash, effervescing drinks, ether, and spirits; all of which are employed with advantage in gastralgias accompanied with vomiting.

(*b.*) M. Lombard has very rarely found vomiting produced by digitalis in those who take much exercise, or whose attention is much occupied during its use. This explains why Orfila and others took twenty or twenty-four grains daily, without its diminishing the frequency of the pulse. If they had remained in bed, it would have been otherwise. It has been observed by many that there are very few accidents with digitalis in those cases where the patient is ignorant of the medicine he is taking. Hence patients should be kept in ignorance, and take as much exercise as their condition will permit.

(*c.*) Dose. As a diuretic, it should be very frequently repeated in the twenty-four hours; but, to quiet palpitations, one grain three or four times daily, or three or four spoonsful of an infusion made with one drachm of digitalis to six ounces of liquid, are sufficient.

(*d.*) The peculiar effects of digitalis on the stomach are produced most easily by the infusion; and they are guarded against in some measure only by combining with it ether and aromatics. For the same purpose, magnesia, sub-nitrate of bismuth, sub-carbonate of iron, and oxide of zinc, are combined with the powder. The subcarbonate of iron is recommended by the Italians, and M. Lombard has found it to be one of the best adjuvants to digitalis; for, by this combination, patients have continued the medicine for many months without inconvenience. It may be given with the infusion. The oxide of zinc is employed by the Germans, and seems to act beneficially by calming an irritable stomach which previously rejected digitalis, not in preventing the symptoms of saturation.

4. *Polygala Senega.* This is a valuable remedy in cases of irregularity of the functions of the heart. Twelve to twenty-four grains of extract, or one drachm of the root infused in four ounces of water, and given daily, appear to diminish the frequency and irregularity of the action of the heart, as well as the consequent sanguineous congestion, in individuals suffering from disease of the heart with dilatation of the cavities.—*Gazette Médicale de Paris*, 10 October, 1835, No. 41.

---

*Creosote.*

AT the sitting of the Academy of Medicine, in Paris, October 5th, M. SOLON made a report, in the names of Caventou and others, on several Memoirs presented to the Academy of Medicine, on the action of Creosote, which called forth a discussion in which the opinions of MM. Andral and Velpeau were elicited. M. Andral had tried it in phthisis, cancer of the uterus, and leucorrhœa, without any results. M. Velpeau had employed it in burns, unhealthy wounds, cancerous, scrofulous, and syphilitic ulcers, and considers it an excitant, or even a mild caustic; but that it is a remedy from which no advantage will be gained, as we have many others of a similar nature, as nitrate of silver, which are infinitely better. The reporters consider it a slight excitant, of no particular value as a medicine, but of use, when diluted, to preserve anatomical preparations.—*Gazette Médicale*, 10 Octobre, 1835, No 41.

---

*Experiments and Observations of the Powers of Creosote on Man and Animals.* By Dr. GUISEPPE CORNELIANI, Professor of Clinical Medicine in the Great Hospital in Pavia.

1. THE internal use of an excessive dose of Creosote produces immediate death without organic lesions.

2. The same thing occurs if it be applied to a large nervous branch, or if it be injected into the veins in minute quantities.

3. If the quantity be not sufficient to produce death, it causes a torpor of sensation and motion, particularly of the lower extremities, the heart, the diaphragm, and the organs of the external senses.

4. As sedatives increase its effects, stimulants would probably relieve them.

5. It produces when taken internally an irritation of the gastro-enteric mucous membrane.

6. Oil and mucilage when combined with it render it milder, but given with vinegar it acts more forcibly.

7. Few patients can bear more than two drops from four to six times in the space of twenty-four hours.

8. Animals poisoned by it pass their urine immediately or soon after death.

9. Applied externally it diminishes irritation, and acts as a desiccator.

10. Applied to ulcers and wounds it promotes cicatrization.

11. When inhaled it causes stupor.

12. It acts as a styptic, provided that the divided vessel be not a large one.

[The experiments were made on lambs, rabbits, dogs, &c.]

*Giornale delle Scienze Medico-Chirurgiche*, No. 8, Febrajo, 1835. Pavia.

